# Alternaria-Induced Release of IL-18 from Damaged Airway Epithelial Cells: An NF-κB Dependent Mechanism of Th2 Differentiation?

**DOI:** 10.1371/journal.pone.0030280

**Published:** 2012-02-07

**Authors:** Hiroki Murai, Huibin Qi, Barun Choudhury, Jim Wild, Nilesh Dharajiya, Swapnil Vaidya, Anjana Kalita, Attila Bacsi, David Corry, Alexander Kurosky, Allan Brasier, Istvan Boldogh, Sanjiv Sur

**Affiliations:** 1 Department of Internal Medicine, University of Texas Medical Branch, Galveston, Texas, United States of America; 2 Division of Pulmonary and Critical Care, Department of Medicine, Baylor College of Medicine, Houston, Texas, United States of America; 3 Department of Biochemistry and Molecular Biology, University of Texas Medical Branch, Galveston, Texas, United States of America; 4 Department of Microbiology, University of Texas Medical Branch, Galveston, Texas, United States of America; Johns Hopkins School of Medicine, United States of America

## Abstract

**Background:**

A series of epidemiologic studies have identified the fungus *Alternaria* as a major risk factor for asthma. The airway epithelium plays a critical role in the pathogenesis of allergic asthma. These reports suggest that activated airway epithelial cells can produce cytokines such as IL-25, TSLP and IL-33 that induce Th2 phenotype. However the epithelium-derived products that mediate the pro-asthma effects of *Alternaria* are not well characterized. We hypothesized that exposure of the airway epithelium to *Alternaria* releasing cytokines that can induce Th2 differentiation.

**Methodology/Principal Finding:**

We used ELISA to measure human and mouse cytokines. *Alternaria* extract (ALT-E) induced rapid release of IL-18, but not IL-4, IL-9, IL-13, IL-25, IL-33, or TSLP from cultured normal human bronchial epithelial cells; and in the BAL fluids of naïve mice after challenge with ALT-E. Both microscopic and FACS indicated that this release was associated with necrosis of epithelial cells. ALT-E induced much greater IL-18 release compared to 19 major outdoor allergens. Culture of naïve CD4 cells with rmIL-18 induced Th2 differentiation in the absence of IL-4 and STAT6, and this effect was abrogated by disrupting NF- κB p50 or with a NEMO binding peptide inhibitor.

**Conclusion/Significance:**

Rapid and specific release of IL-18 from Alternaria-exposed damaged airway epithelial cells can directly initiate Th2 differentiation of naïve CD4^+^ T-cells via a unique NF-κB dependent pathway.

## Introduction

Asthma is one of the most common health afflictions worldwide. Approximately 300 million people suffer from asthma, and 70% of whom have associated allergies [1]. The airway epithelium is the first line of defense against inhaled allergens. Recently, a large-scale, consortium-based genomewide association study of over 10,365 persons with physician-diagnosed asthma and about 16000 unaffected control subjects strongly implicated an important role of epithelial damage in activation of the adaptive immune system and induction of allergic airway inflammation and asthma [2]. However, relatively little is known about specific environmental factors that induce epithelial damage and cytokine release that promote Th2 differentiation and allergic asthma.

Large multicenter studies have evaluated the relationship between allergic sensitization to outdoor allergens and asthma [3]–[5]. The Childhood Asthma Management Program (CAMP) study of over 1000 children investigated the relationship between sensitization to inhalant allergens, such as *Penicillium*, *Aspergillus*, Timothy, Bermuda grass, short ragweed and *Alternaria*, lung function and bronchial responsiveness. Sensitization to only *Alternaria* was associated with bronchial hyperresponsiveness (p<0.01) [5]. Similarly, in a study of 895 children that examined the association between asthma and sensitization to allergens such as Timothy, Bermuda, Ragweed, Tree mix, *Alternaria*, *Penicillium*, *Aspergillus*, allergy to only *Alternaria* was associated with increased risk for asthma at ages 6 and 11 [6]. In the NHANES II study, 5000 persons 6 to 74 years age were tested for allergy to *Alternaria*, mixed giant and short ragweed, oak, perennial ryegrass and Bermuda grass outdoor allergens; asthma was associated with allergy to only one outdoor allergen, *Alternaria* [4]. Finally in the NHANES III study, 12,000 subjects 6 to 59 years were tested for allergy to perennial rye grass, short ragweed, Bermuda grass, Russian thistle, white oak and *Alternaria*; allergy to *Alternaria* was associated with asthma [3]. Together, these studies performed in about 21,000 children and adults have reproducibly shown that sensitization to *Alternaria* is a key outdoor allergen associated with asthma. However, to date, the molecular basis of this association remains a scientific enigma.

The airways in mice and humans contains epithelial and dendritic cells (DCs) that are the first cells to respond to inhaled allergens [7], [8]. Prior studies have demonstrated the presence of intraepithelial class II major histocompatibility complex antigen (Ia)-bearing dendritic cells (DC) in the conducting airways [9]–[12]. These airway DCs have emerged as key cells that initiate CD4+ T-cell responses that direct Th2 response in vivo [8], [13]–[15]. The airway epithelium can produce several cytokines such as thymic stromal lymphopoietin (TSLP), IL-25 and IL-33 that play a critical role in induction of Th2 differentiation, nuocyte formation and induction of allergic asthma [16]–[20]. The effects of TSLP and IL-25 require STAT6 and IL-4, and both cytokines work synergistically to promote Th2 differentiation [17], [19], [20]. However, the normal airway epithelium is a powerful barrier against the development of antigen-specific Th2 cells and allergic airway inflammation [21]–[23]. We hypothesized that *Alternaria* is a unique allergen that rapidly induces damage to the epithelium, releasing cytokines that promote Th2 differentiation of naïve T-cells.

In this report, we show that *Alternaria* extract induces damage to the airway epithelium, selectively and rapidly releasing IL-18 but not other Th2-associated cytokines such as IL-4, IL-9, IL-13, IL-25, IL-33, and TSLP from cultured normal human bronchial epithelial cells (NHBE) cells, and in the airways of naive mice. We also show that rIL-18 by itself is sufficient to induce Th2 differentiation.

## Results

### *Alternaria* extract, but not other outdoor allergen extracts, rapidly induces IL-18 release from airway epithelial cells

**Figure 1:**
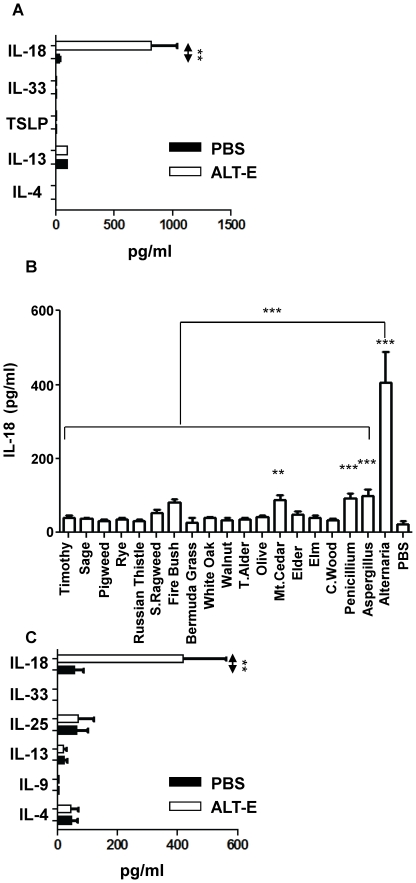
Rapid release of cytokines from epithelial cells cultured with ALT-E. (**A, B**) NHBE cells were cultured with PBS, ALT-E or other allergens for 15 min, and released cytokines were quantified. T. Alder, Tag Alder; S. Ragweed, Short Ragweed (n = 5 wells per treatment group). (**C**) BALF cytokine levels in naïve mice 60 min after challenge with 20 µg ALT-E or PBS control (n = 4 per group).

Since airway epithelial cells are the first barrier against inhaled allergens, we first focused our efforts on identifying Th2-inducing cytokines secreted from Normal Human Bronchial Epithelial (NHBE) cells culture with ALT-E. NHBE cells were cultured for 15 minutes with Alternaria extract (ALT-E), and the cell supernatants were examined for IL-4, IL-13, IL-18, IL-33 and TSLP. ALT-E rapidly induced a 26-fold increase in IL-18 levels; other Th2-associated cytokine levels were not significantly alterted (**Fig. 1A**). Furthermore, ALT-E (30 µg/ml) induced much greater IL-18 release from NHBE cells compared to same concentration of 19 major outdoor allergens that were tested in multicenter epidemiologic studies [3]–[5], demonstrating a strong specificity of release (**Fig. 1B**). To determine the in vivo relevance of IL-18 release from NHBE cells cultured with ALT-E, we examined the innate effects of intrapulmonary administration of ALT-E in naïve mice. Similar to the observations in NHBE cells, ALT-E challenge induced a 8.6-fold increase in IL-18 levels in BAL fluid 1 hr post challenge (**Fig. 1C**) relative to PBS control animals. These data indicate that ALT-E induces specific release of IL-18 from epithelial cells.

### *Alternaria* extract rapidly induces epithelial cell necrosis

**Figure 2:**
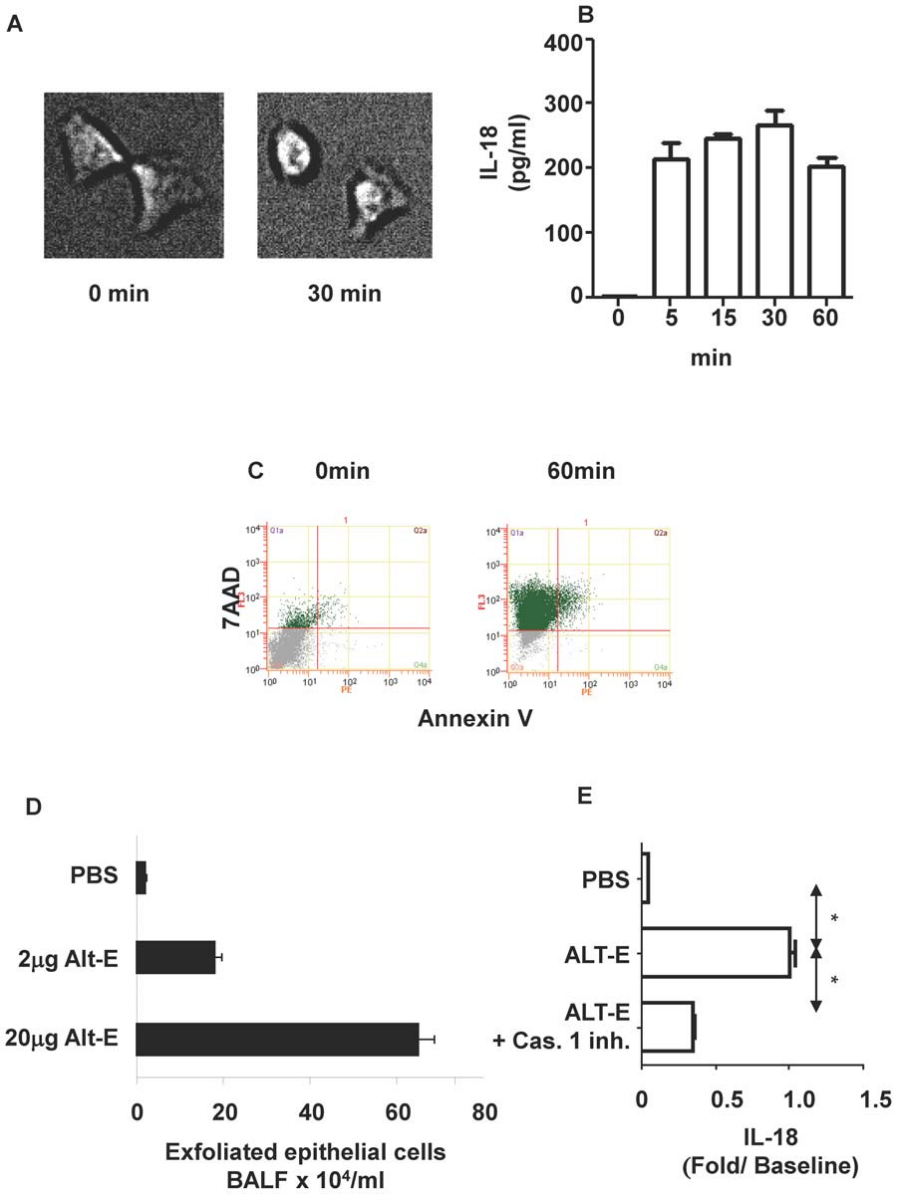
Induction of epithelial cell necrosis by ALT-E. (**A**) A549 cells were cultured with ALT-E for 30 min. Changes in cell morphology were monitored by live cell imaging. (**B**) Time course of ALT-E-induced IL-18 release from airway epithelial cells. (**C**) Exposure of cultured A549 epithelial cells to ALT-E induces cell necrosis. (**D**) ALT-E challenge of mice induces sloughing of trypan blue positive necrotic airway epithelial cells (n = 4 per group). (**E**) Caspase 1 inhibitor (Cas.1 inh.) reduces IL-18 release from A549 airway epithelial cells cultured with ALT-E.

Next we examined the mechanism of this release of IL-18 from epithelial cells. Incubation of A549 bronchial epithelial cells with ALT-E (30 µg/ml) rapidly induced swelling of cells. The cells became non-adherent, and rounded in 30 min. (**Fig. 2A**). ALT-E also induced similar swelling and rounding of NHBE cells (data not shown). During this early time the cells rapidly released IL-18 as early as 5 min; release plateaued thereafter (**Fig. 2B**). Release of IL-18 was not inhibited in the presence of broad spectrum protease inhibitors (data not shown). Furthermore, other allergens with high protease activity allergens, such as house dust mite and American cockroach extracts, failed to induce IL-18 release (data not shown). Additionally, release of IL-18 was independent of ROS because adding anti-oxidants along with Alt-E failed to decrease IL-18 release (data not shown). We observed that ALT-E increased cell permeability for 7AAD (a necrotic cell surface marker), but the cells poorly bound Annexin V (an apoptosis marker) (**Fig. 2C**), indicating that it induces necrosis of epithelial cells. To determine the in vivo relevance of these observations, naïve mice were challenged with ALT-E; this challenge rapidly induced exfoliation of dead epithelial cells from the lungs of mice within 30 mins (**Fig. 2D**). Building on this observation and because necrosis of cells has been shown to activate inflammasomes [24], we examined whether IL-18 release was dependent on caspase 1. The inhibitor caspase 1 reduced IL-18 release (**Fig. 2E**). Together these studies indicate that ALT-E induces release of IL-18 from airway epithelial cells by activating caspase 1 and inducing necrosis.

### IL-18 induces Th2 differentiation of naïve CD4^+^ T cells independent of IL-4 and STAT6

**Figure 3:**
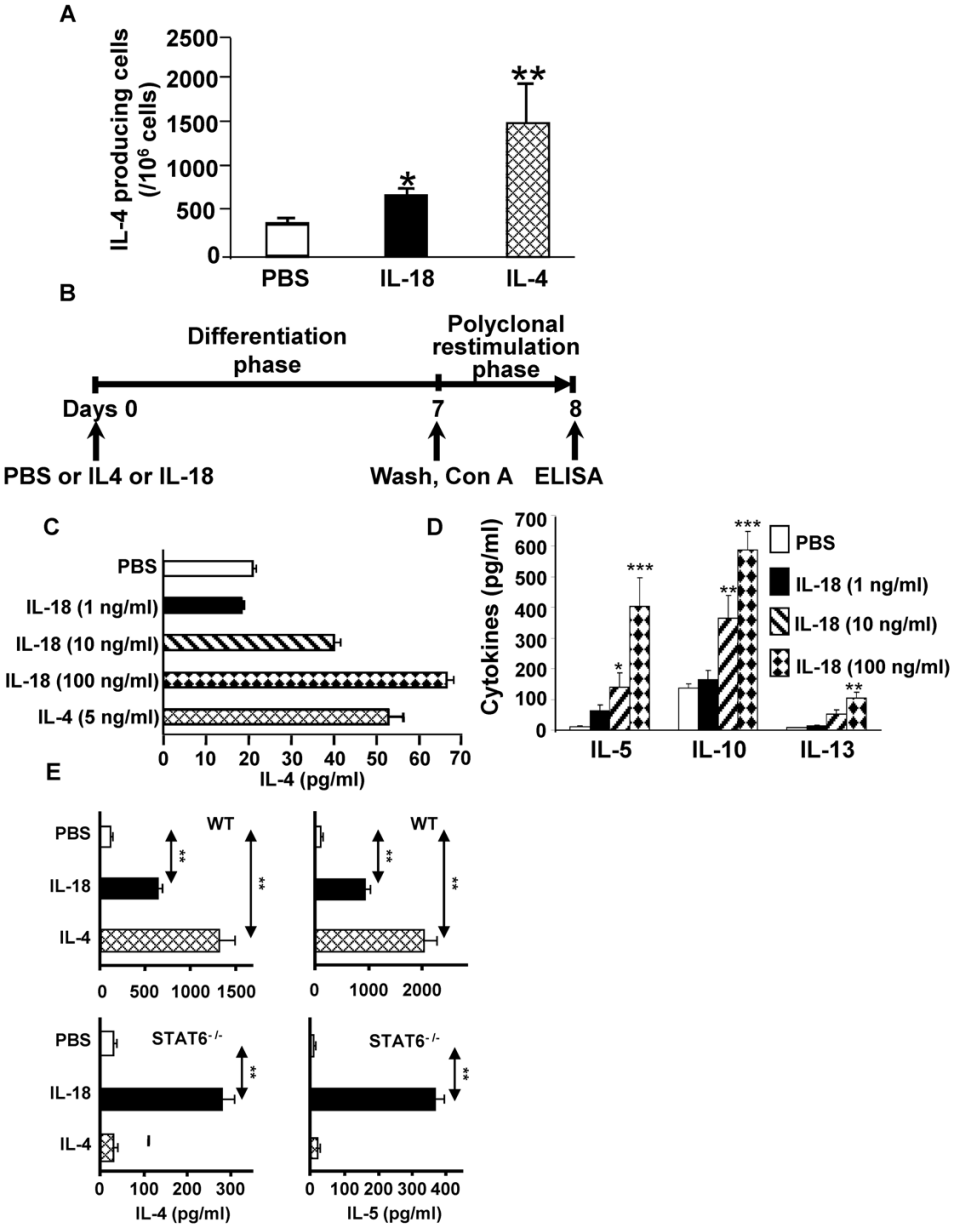
Effect of IL-18 on induction of Th2 differentiation. (**A**) Effect of IL-18 on IL-4 secreting splenocytes ELISPOTS. (**B**) T cell differentiation protocol used in Panels 3C–E and Fig. 4. (**C, D and E**) Effect of IL-18 or IL-4 on Th2 differentiation of naïve CD4+ T-cells isolated from WT mice (**C**), IL-4^−/−^ mice (**D**) and WT and STAT6^−/−^ mice (**E**). n = 5−7 per group.

The intrinsic protease activity of ALT-E and its ability to induce extracellular ATP can have direct effects on dendritic and T-cells to induce Th2 differentiation [25]–[27]. Since our objective was to determine the Th2-promoting effects of IL-18 that is rapidly and specifically released from epithelial cells upon contact with ALT-E, we designed experiments that did not require direct contact of ALT-E with immune cells. This was achieved by adding rIL-18 to purified naïve CD4+ T-cells and examining whether it could provide sufficient signal required to induce Th2 differentiation. Because CD4^+^ T cells require interaction of IL-4 with IL-4R and activation of STAT6 to induce Th2 differentiation [28], [29], we initially examined whether IL-18 could promote Th2 differentiation by inducing secretion of IL-4. Indeed treatment of naïve splenocytes with either 50 ng/ml IL-4 or 100 ng/ml of IL-18 for 8–10 hours rapidly increased the number of IL-4 secreting cells (**Fig. 3A**). Next, we examined the ability of IL-18 to induce Th2 differentiation of naïve CD4^+^ T cells (**Fig. 3B**). As expected, culture of CD4^+^ T cells with plate-bound anti-CD3 and anti-CD28 in the presence IL-4 induced Th2 differentiation (**Fig. 3C**). Likewise, IL-18 also induced Th2 differentiation of CD4^+^T cells in a dose dependent manner (**Fig. 3C**). Taken together, the data in **Fig. 3A–C** suggest that IL-18 induces Th2 differentiation of naïve CD4^+^ T-cells.

To examine the role of secreted IL-4 in mediating the Th2 differentiating effects of IL-18, CD4^+^ T cells from IL-4^−/−^ mice were cultured with IL-18. IL-18 induced Th2 differentiation of CD4^+^ T cells from IL-4^−/−^ mice (**Fig. 3B**), as indicated by the secretion of IL-5, IL-10, IL-13 (**Fig. 3D**). These observations indicate that IL-18 induces Th2 differentiation independent of IL-4. Since STAT6 is required for inducing Th2 differentiation [28], [29], we examined the role of STAT6 in mediating IL-18 in inducing Th2 differentiation. CD4^+^ T cells derived from WT mice and STAT6^−/−^ mice were cultured with IL-18. As expected, culture of STAT6^+/+^ T-cells with 50 ng/ml of IL-4 in the differentiation phase induced IL-4 and IL-5 release upon polyclonal restimulation, and these effects were abrogated by disruption of STAT6 (**Fig. 3E**). Likewise, culture of STAT6^+/+^ CD4^+^ T-cells with IL-18 in the differentiation phase induced IL-4 and IL-5 release upon polyclonal restimulation. Surprisingly, these effects of IL-18 were not completely abrogated by disruption of STAT6 (**Fig. 3E**). These results indicate that IL-18 can promote Th2 differentiation of naïve CD4^+^ T cell even in the absence of IL-4 and STAT6.

### IL-18 induces Th2 differentiation via NF-κB

**Figure 4:**
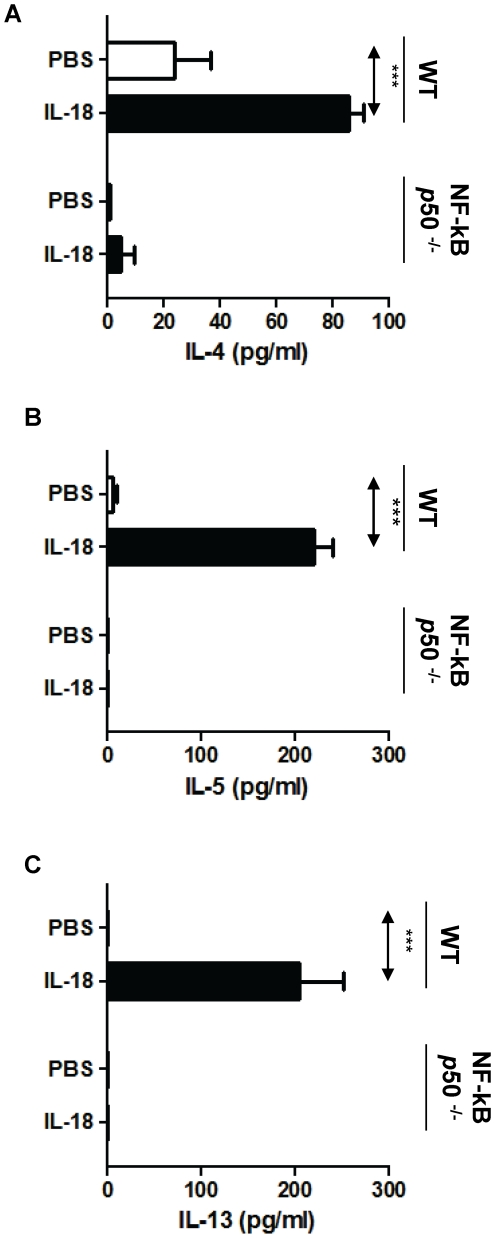
Effect of disruption of NF-κB p50 on Th2 differentiation of naïve CD4 T-cells treated with IL-18. CD4^+^ T-cells from Wild-type (WT) and NF-κB p50^−/−^ mice were cultured with IL-18 in the differentiation phase, restimulated with con A, and secretion of IL-4 (**A**), IL-5 (**B**) and IL-13 (**C**) was quantified by ELISA. n = 5−7 per group.

**Figure 5:**
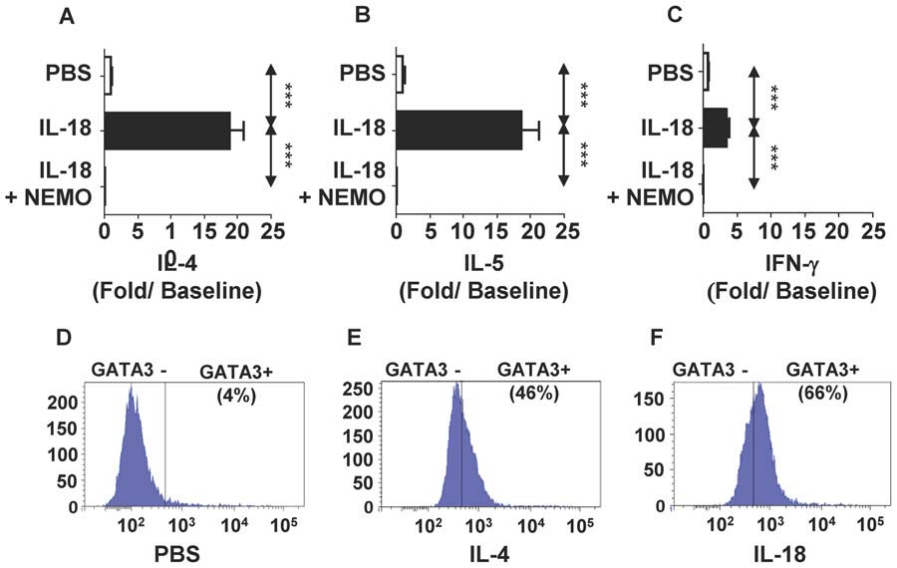
Effect of IL-18 on Th2 differentiation and GATA3 expression in naïve CD4+ T-cells. (**A,B,C**). Effect of NEMO peptide on Th2 differentiation. Negatively selected naïve CD3^+^ CD4^+^ T-cells were cultured with plate bound anti-CD3 and anti-CD28 with PBS (PBS), IL-18 100 ng/ml (IL-18), IL-18 100 ng/ml and 10 µM NEMO Binding Domain Binding Peptide (IL-18+NEMO) for the initial culture period. The cells were washed and restimulated, and secretion of IL-4 (**A**), IL-5 (**B**) and IFN-γ(**C**) were quantified. The data was expressed as fold increase compared to cells cultured with PBS. (**D,E,F**). GATA3 expression in T-cells. Negatively selected naïve CD3^+^ CD4^+^ T-cells were cultured with plate bound anti-CD3 and anti-CD28 in the presence of PBS (**D**), IL-4 (**E**) or IL-18 (**F**) for 7 days. GATA3 expression was measured by flow cytometric analysis.

Prior studies have shown that IL-18 signals through MyD88 [30] and activates NF-κB [31]. Furthermore, NF-κB p50 plays a critical role in allergic sensitization and asthma [32]; however, to our knowledge, Th2 differentiation of CD4^+^ T cells via NF-κB has not been described. Here we found that culture of naïve T-cell derived from WT mice with IL-18 under non-skewing conditions induced a significant increase of IL-4 (**Fig. 4A**), IL-5 (**Fig. 4B**), and IL-13 (**Fig. 4C**), indicating Th2 differentiation. These effects of IL-18 were abrogated in NF-κB p50^−/−^ mice. Because disruption of NF-κB p50 could alter T-cells in unforeseen ways that could explain these observations, we wished to confirm our observations in T-cells from wild-type mice using a specific inhibitor of the NF-κB pathway. To achieve this objective, we compared the evaluated the effect of culture of CD4+ T-cells in the presence or absence of NEMO-Binding Domain Binding Peptide (**Fig. 5 A–C**) in a T-cell differentiation assay. In the absence of NEMO inhibitor, IL-18 vigorously increased (20-fold) IL-4 and IL-5 secretion (**Fig. 5A** **and** **Fig. 5B**) from restimulated cells; these effects were inhibited by NEMO peptide inhibitor. As the experiments were performed under non-skewing conditions, it is not surprising that IL-18 treatment also induced a modest 3-fold increase in IFN-γ secretion (**Fig. 5C**). Importantly, IL-18 treatment increased GATA3 expression in 66% of CD3^+^CD4^+^ T-cells (**Fig. 5F**). Taken together, these results indicate that IL-18 induces Th2 differentiation via a novel NF-κB p50-dependent pathway.

## Discussion

Multicenter epidemiologic studies such as CAMP, NHANES II and III have reproducibly shown that *Alternaria* sensitization is associated with allergic asthma, but the mechanism of this specific association remains a scientific enigma [3]–[6]. Here we show that exposure of NHBE cells and the airways of naïve mice to ALT-E rapidly induces the release of IL-18, but not other Th2 associated cytokines such as IL-4, IL-9, IL-13, IL-25, IL-33 or TSLP. Furthermore, when compared to 19 other major outdoor allergens examined in these multicenter epidemiologic studies, *Alternaria* extracts released the greatest amounts of IL-18 from airway epithelial cells than the other outdoor allergens. Importantly, release of IL-18 was associated with damage to these airway epithelial cells. The GABRIEL consortium study predicted environmentally induced epithelial damage to activate the adaptive immune system and induce allergic airway inflammation and asthma [2]. Our results suggest that *Alternaria* exposure damages the airway epithelium and releases IL-18, and this release may be one of the mechanisms of initiation of asthma. Furthermore, the consortium study identified protective effects of single nucleotide polymorphisms (SNPs) in IL-18R against asthma, suggesting pro-asthma effects of wild-type IL-18R, which is also consistent with our study [2].

Our observations indicate that airway epithelial cells release IL-18 within minutes of exposure to ALT-E, and this release was associated with epithelial cell necrosis. The protease activity of fungi has been shown to induce Th2 inflammation [25], [26], [33] and increase TSLP secretion via PAR2 receptor in human BEAS2B cells in 12 hrs [25]. Another recent report suggested that *Alternaria* induces release of IL-33 into the murine airway lumen, followed by Th2-type responses [27]. However, in our study that utilized physiologically relevant human NHBE cells, ALT-E increased IL-18 but not TSLP or IL-33 levels in 15 mins. The much shorter time frame in our study and the various epithelial cells studied may account for the differences in these studies. It is unlikely that the protease activity of ALT-E contributed to IL-18 release in our study because broad spectrum protease inhibitors failed to inhibit IL-18 release, and high protease allergens such as American cockroach or house dust mite did not increase its secretion. The more likely explanation for IL-18 release in our study is that ALT-E induces rapid necrotic change in epithelial cells. Necrotic cell death has been shown to activate the NLRP3 inflammasomes that convert pro-IL-18 to IL-18 by activating caspase 1 [24]. Our observations indicate that an inhibitor of caspase 1 decreases levels of IL-18 release. Future studies should carefully evaluate whether inflammasome or other mechanisms play a role in Alt-E induced rapid release of IL-18 from airway epithelial cells.

A prior report indicated that IL-18 promotes Th2 differentiation by inducing secretion of IL-4 [34]. Consistent with that report, we show here that IL-18 induced IL-4 secretion from T-cells. However, our results also indicate that IL-18 can induce Th2 differentiation independent of IL-4. Since both studies utilized similar doses of IL-18 to stimulate T-cells, these two different observations cannot be rationalized by differences in IL-18 dose used in the two studies. In the prior study, the Th2 differentiation promoting effects of IL-4 were evaluated by using 11B11 neutralizing antibody against IL-4 [34]. In contrast, here we utilized a more rigorous approach by using CD4^+^ T cells from IL-4^−/−^ mice. It is possible that this difference in extent of IL-4 blockade in the two studies could account for the difference in final observations.

In the present study, we show that IL-18 can induce Th2 differentiation even in the absence of STAT6. These observations were very surprising because elegant studies have reported that IL-4 and STAT6 are indispensable for inducing Th2 differentiation [28], [29]. T cells lacking IL-4 and Stat6 have been shown to express higher levels of IL-18Rα after T-cell Receptor (TCR) stimulation, a key driving factor for Th1 induction [35]. However, the mechanism of Th2 differentiation is unlikely to be due to downregulation IL-18Rα expression by IL-4 because we show Th2 differentiation in the absence of IL-4 [35]. Because GATA3 expression in the absence of STAT6 is sufficient to induce Th2 differentiation [36], and our data in the present study indicates that IL-18 can increase GATA3 expression, the most likely mechanism of th2 induction is increased GATA3 expression.

Previous reports indicated that mice deficient in the p50 subunit of NF-κB show reduced eosinophilic airway inflammation compared with wild-type mice. This deficiency was not due to a block in T cell priming or proliferation in the p50^−/−^ mice [32], [37]. Here we show that the Th2 differentiating effects of IL-18 are inhibited in the absence of NF-κB p50, or by inhibiting NF-κB pathway in wild type T-cells using a NEMO binding peptide inhibitor. It is consequently likely that in vivo Th2 promoting effects of IL-18 will be blocked by inhibitors of this pathway.

In the present study, IL-18 induced a slight increase in IFN-γ secretion from restimulated CD4 cells. Because these experiments were performed under non-skewing conditions, without using neutralizing antibodies against IFN-γ or IL-12, these culture conditions likely account for this slight increase in IFN-γ. These observations are consistent with our earlier work showing that intrapulmonary administration of IL-18 with an allergen in mice promotes allergic sensitization and Th2 phenotype, and IFN-γ induction by IL-18 has short-term inhibitory effects on allergic inflammation that disappears by three weeks of administration, whereas the Th2 effects become dominant after three weeks and are long lasting [38].

In summary, we provide evidences that exposure to an extract of *Alternaria alternata* has the ability to rapidly induce epithelial cell necrosis and specifically release IL-18. This effect of Alternaria on epithelial cells may involve conditioning of DC [39], [40], and the released IL-18 is likely to contribute to the development of allergic asthma by its ability to induce Th2 differentiation by a novel NF-κB-dependent pathway. We suggest that these molecular pathways could be one of the pathways that link epithelial cell damage to asthma as hypothesized in the GABRIEL consortium study [2], and explain the reproducible association between *Alternaria* sensitization and allergic asthma in the CAMP and NHANES studies.

## Materials and Methods

### Cell cultures and treatments

Primary normal human bronchial epithelial (NHBE) cells (Cambrex Bio Science, Walkersville, Maryland) were cultured in BEGM® BulletKit® medium supplied by the manufacturer (Lonza, Switzerland). NHBE cells were maintained as previously described [41]; 2–4 passages of NHBE cells were used for this study. A549 bronchial epithelial cells were obtained from the American Type Cell Collection and cultured in Ham's F-12 medium at 37°C in a humidified atmosphere with 5% CO_2_. For experiments cells were subcultured into 96 well plates and exposed to 30 µg/ml of various endotoxin-free allergens for 15 min; supernatants were collected and analyzed using cytokine ELISA assays. Caspase-1 inhibitor I (Calbiochem, Gibbstown, NJ), 10 uM, was added to 1×10^5^ A549 cells for 1 hr before exposure to 30 µg/ml of ALT-E stimulation.

### Allergens

The allergens tested were the identical outdoor allergen panel that was examined in the NHANES and CAMP studies, consisting of *Alternaria*, *Aspergillus*, *Penicillium*, Cotton wood, Elm, Elder, Mountain Cedar, Olive, Tag Alder, Walnut, White Oak, Bermuda Grass, Fire Bush, Short Ragweed, Russian thistle, Perennial rye grass, Pigweed, Sage grass and Timothy grass (Greer, NC, USA).

### Live cell imaging for cell necrosis

Morphologic changes were continuously monitored and recorded at 37°C in 5% CO_2_ using NIKON live-cell imaging microscopy (NIKON Eclipse TE200).

### Flow cytometry for cell necrosis and apoptosis

A549 cells were cultured with 30 µg/ml allergen extract at 37°C in 5% CO_2_ and analyzed for cell necrosis and apoptosis using Annexin-V Apoptosis detection kit 1 (BD Pharmingen, San Diego, CA) according to company's protocol. A minimum of 15,000 cells were analyzed using Beckman Coulter Quanta for FACS.

### Animals

Eight weeks old BALB/c mice purchased from Harlan Sprague Dawley (Indianapolis, IN). Stat6^−/−^ and IL-4^−/−^ on BALB/c background were purchased from Jackson laboratory (Bar Harbor, Maine). The NF-κB p50^−/−^ mice and their controls were purchased from the Jackson Laboratory. All mice were housed in a pathogen-free environment throughout the experiment at The University of Texas Medical Branch at Galveston. The Institutional Animal Care and Use Committee of The University of Texas Medical Branch at Galveston approved all animal experiments (IACUC Approval ID: 9708038A).

### Bronchoalveolar lavage (BAL)

Bronchoalveolar lavage was performed as previously described [42], [43]. Mice were euthanized with an intraperitoneal injection of ketamine and xylazine, and challenged intranasally with 20 µg/ml of ALT-E, or PBS. After 1 hr, the BALF were obtained by cannulating the trachea and lavaging the lungs with two 0.75 ml aliquots of ice-cold Dulbecco's PBS (Sigma, St. Louis, MO). The cells in BAL fluids were pelleted and the number of trypan blue positive ciliated epithelial cells were quantified by counting cells in a hemocytometer. The cytokines levels in cell-free BAL fluids were quantified by ELISA.

### Purification of naïve CD4^+^ T cells

Spleen were dissected from naïve mice, washed with PBS twice, and separated into cells using 70 µm cell strainer (BD falcon, Sanjose CA). T cells were negatively purified with T cell Isolation kit II and Auto MACS separater (Militenyi Biotech, Germany, >95% pure).

### ELISA for cytokines

Two-site immunoenzymetric assays were used to measure IFN-γ, IL-4, IL-9, IL-13, IL-18, IL-25, IL-33, and TSLP. Human IL-18 was measured using capture antibody clone number 159-12B, and the detection antibody clone 125-2H (R and D Systems). Human IL-4, IL-13, IL-33 and TSLP were measured by ELISA Kits (R&D Systems, Minneapolis, MN). Murine IL-4, IL-9, IL-10 and IL-18 were measured using capture antibody clone numbers 11B11 (IL-4), D8402E8 (IL-9), JES5-2A5 (IL-10); and the detection antibody clone numbers were BVD6-24G2 (IL-4), D9302C12 (IL-9), JES5-16E3 (IL-10) (all purchased from BD Biosciences), and IL-18 capture antibody clone 74 and detection antibody clone 93–10 C (R and D Systems). Mouse IL-13, IL-25, IL-33 and TSLP were measured by DuoSet Kits (R&D systems, Minneapolis. MN).

### ELISPOT for IL-4-secreting cells

ELISPOT for IL-4 secreting cells were undertaken as previously described [43]. Briefly, Immulon 2 microtiter plates (96-well) were coated with anti-IL-4 (clone BVD4-1D11, Endogen, Woburn, MA). Serial dilutions of spleen cells, ranging from 1×10^5^ to 10×10^5^ cells/well, were incubated on anti-cytokine-coated plates for 8–10 h at 37°C in a humidified 5% CO_2_ incubator. Plates were then washed and overlaid with biotinylated anti-IL-4 (clone BVD6- 24G2, Endogen), washed, and treated with a 1∶2000 dilution of avidin conjugated alkaline phosphatase (Vector Laboratories, Burlingame, CA) for 2 hrs at room temperature. After a final wash, the cytokine products of individual secreting cells were visualized by the addition of a solution of 5-bromo-4-chloro-3-indolyl phosphate/nitroblue tetrazolium (Kirkegaard and Perry, Gaithersburg, MD). ELISPOTs were counted with the aid of a dissecting microscope and expressed as ELISPOTs per million cells.

### Th2 differentiation assays

Briefly, negatively selected Naïve CD4^+^ T cells (>95% pure, negatively selected) were cultured in wells precoated with 1 µg/ml anti-CD3 and anti-CD28 for 7 days with a range of IL-18 (1–100 ng/ml) and an IL-4 concentration of 1 ng/ml. IL-2 (50 U/ml) was added to all wells after 24 hr. The cells were extensively washed and restimulated with Con A for 24 hr. The supernatants were analyzed IL-4, IL-5, and IL-13 by ELISA.

### Th2 differentiation assays with NEMO-Binding Domain Binding Peptide inhibitor

Negatively selected murine CD4+ T cells were cultured with plate bound anti-CD3 and anti-CD28 as outlined (see Fig. 3B above) along with PBS, or IL-18 (100 ng/ml) or IL-18+NEMO-Binding Domain Binding Peptide (NEMO, 10 µM) and subjected to analysis outlined above.

### Flow cytometric analysis for GATA3 expression in CD4+ T cells

CD4^+^ T-cells were cultured with plate bound anti-CD3 and anti-CD28 along with PBS, IL-4 20 ng/ml, or IL-18 (100 ng/ml) for 7 days. The cells were harvested and subjected to flow cytometric analysis for GATA3 expression. CD3+ (FITC) CD4+ (PE-Cy5) T-cells were gated and analyzed for GATA3 expression shown on the X-axis. All FACS antibodies were purchased from BD Biosciences, San Diego, CA.

### Statistics

Data from different treatment groups were analyzed by ANOVA, followed by Bonferroni post-hoc analyses for least significant difference. Values in figures are expressed as mean ± SEM. *P<0.05,**P<0.01,***P<0.001.
